# ETHICAL CHALLENGES THAT ARISE AT THE COMMUNITY INTERFACE OF HEALTH RESEARCH: VILLAGE REPORTERS' EXPERIENCES IN WESTERN KENYA

**DOI:** 10.1111/dewb.12023

**Published:** 2013-03-22

**Authors:** Tracey Chantler, Faith Otewa, Peter Onyango, Ben Okoth, Frank Odhiambo, Michael Parker, Paul Wenzel Geissler

**Keywords:** Africa, developing world bioethics, empirical ethics, clinical trials, community engagement, relational ethics, vulnerable populations

## Abstract

Community Engagement (CE) has been presented by bio-ethicists and scientists as a straightforward and unequivocal good which can minimize the risks of exploitation and ensure a fair distribution of research benefits in developing countries. By means of ethnographic fieldwork undertaken in Kenya between 2007 and 2009 we explored how CE is understood and enacted in paediatric vaccine trials conducted by the Kenyan Medical Research Institute and the US Centers for Disease Control (KEMRI/CDC). In this paper we focus on the role of paid volunteers who act as an interface between villagers KEMRI/CDC. Village Reporters’ (VRs) position of being both with the community and with KEMRI/CDC is advantageous for the conduct of trials. However it is also problematic in terms of exercising trust, balancing allegiances and representing community views. VRs role is shaped by ambiguities related to their employment status and their dual accountability to researchers and their villages. VRs are understandably careful to stress their commitment to self-less community service since it augments their respectability at community level and opens up opportunities for financial gain and self-development. Simultaneously VRs association with KEMRI/CDC and proximity to trial participants requires them to negotiate implicit and explicit expectations for material and medical assistance in a cultural setting in which much importance is placed on sharing and mutuality. To ensure continuity of productive interactions between VRs, and similar community intermediaries, and researchers, open discussion is needed about the problematic aspects of relational ethics, issues concerning undue influence, power relations and negotiating expectations.

## Introduction

Community participation in promoting and sustaining health was championed in the Declaration of Alma Ata on Primary Health Care.^1^ This declaration stated: ‘the people have the right and duty to participate individually and collectively in the planning and implementation of their health care’. Community participation and self-reliance were stressed as invaluable to achieving sustainable development. The Alma Ata recommendations were widely adopted in developing countries by policymakers, health professionals, funders and communities, and implemented to differing degrees with varying success.^2^

The involvement of community members under various names such as village health helpers or community health workers was integral to Alma Ata and subsequent initiatives prioritised the training and equipping of health volunteers. Despite a move away from community-led health programmes during the late 1980s and 1990s, current government health strategies, such as the one adopted by Kenya are actively incorporating such volunteers, in health education and primary health care activities.^3^

Over the past decade community members have become increasingly involved in health research taking place in developing countries, both as volunteers and contracted employees. One of their primary responsibilities is to act as intermediaries between lay people and scientists. The involvement of such community intermediaries reflects the increased attention paid to community engagement (CE) as means of protecting communities against exploitation.^4^ In a framework developed by Emmanuel et al.,^5^ collaborative partnerships between researchers, health policymakers and the community are conceived as a way of ensuring that research is ethical. In practice, CE is defined as: ‘a process of working collaboratively with relevant partners, who share common goals and interests’.^6^

In this paper we examine an ‘ethics’ based on relationships, attachment and familiarity. Respectful relationships are core to the Emanuel et al.^7^ framework and their reference to benchmarks for measuring good practice has initiated broader discussion about ethics and community. It is thought that overreliance on formal guidance, principles and a related ‘tick box’ mentality can stifle ethical reflection. Geissler et al.^8^ argue that research ethics should make space to unfold ethical relations which either pre-exist or develop in the implementation of public health trials. Drawing on ethnographic research on interactions between community members and research assistants who were based in villages hosting vaccine research, they highlight the importance of attachment and familiarity versus detachment. Whilst attachment made it difficult for the research assistants to uphold certain trial restrictions (e.g., medication being available only to trial participants), the formation of social bonds allowed their interactions to be guided by their ethical impulse or moral compass. These types of interactions which are characterised by increasing familiarity and trust need to be considered more carefully in the application of ethical guidelines.

Trust is a relational notion which describes a voluntary relationship between two or more people (inter-personal trust) or between a person and an institution (institutional trust).^9^ Molyneux and colleagues demonstrate its importance with particular reference to consent and community perceptions of research.^10^ Their work emphasises the need to understand the social context and ensure that research teams incorporate both technical and inter-personal competence. The latter may be achieved by working with community -based research assistants who are known and trusted by local residents and can serve as community intermediaries and cultural brokers for researchers.^11^ Whilst the advantages of such community intermediaries are evident some attention has been drawn to ethical considerations. Simon & Mosavel's^12^ concern is the potential for ‘vertical exploitation’ which can occur when outside researchers exploit community intermediaries’ social connections with local community members to promote research. Specific reference is made to recruitment practices, but ‘vertical exploitation’ also covers potentially unfair employment practices. *‘*Horizontal exploitation’, by contrast, is described to occur when community intermediaries exploit their partnerships with outside researchers to gain power and influence within their communities.^13^ These duel ethical concerns demonstrate that power relations have to be taken seriously and thought about carefully in the conduct of health research and related CE.

All international public health research involves fieldwork (e.g. data collection, participant recruitment and consent) which is usually undertaken by different types of community-based research assistants. One key differential lies in their employment status which can range from contracted staff to volunteers who derive certain monetary benefits from their involvement in research. Furthermore formally employed community-based research assistants need to satisfy pre-determined skills and educational criteria, whereas volunteers are usually selected by a nomination process which involves community members. Our experience suggests that nominees are those: whose knowledge, experience and age is respected; whose association with a faith group gives them moral standing; or whose activities promote local investment and social good.

This paper documents the experiences of Village Reporters (VRs) who support research conducted by the Kenya Medical Research Institute in collaboration with the US Centres for Disease Control and Prevention (KEMRI/CDC). VRs’ perspectives are of interest since they reside permanently in the villages where they work, and so must balance kinship, cultural and professional boundaries. Unlike other community-based research assistants, they are casual workers and not contracted employees, so they are not as closely accountable to researchers. VRs are comparable to other casual employees (e.g. peer recruiters) engaged as community intermediaries in similar settings in the developing world. This provides additional justification for documenting their experiences and considering the practical and ethical implications.

## Methodology

This research formed part of an ethnographic study exploring how CE is practised in vaccine trials conducted by KEMRI/CDC. The aim was to analyse the relationship between CE and ethical practice in paediatric vaccine research. In this paper we concentrate on the role of VRs who are central to the comprehensive CE strategy at KEMRI/CDC. At the time of our fieldwork three clinical trials had reached different stages in testing the effectiveness and efficacy of three paediatric vaccines. We refer to these trials as Trial A, B and C, and do not elaborate on the candidate vaccines.

Our fieldwork was conducted by a team of international and national researchers between October 2008 and August 2010. TC coordinated the work, undertook participant observations, and conducted interviews supported by two local assistants. The latter and PO spoke fluent Dholuo (local language) and served as cultural brokers, transcribers and translators.

Purposive sampling^14^ was used to identify information-rich cases. Potential participants were given a letter outlining the purpose of the study and those who expressed interest were later interviewed after giving informed consent. The primary data used for this paper comprise interviews and group discussions held with 9 VRs and 18 researchers. To support ethnographic description we also draw on participant observations of trial-related and community activities, household visits by VRs, VR meetings with researchers, source documents, and insights gained from interviews and focus groups with community advisory board members (n = 17), parents of trial participants (n = 27), community members and leaders (n = 48).

Transcripts were imported into a qualitative data software programme (NVivo 8) to facilitate the development of a coding framework. Data analysis was thematic, and field notes provided both contextual and thick description. The analysis was undertaken by TC and verified by the other authors.

Ethical approval was obtained from the KEMRI ethics review committee (#1302), CDC Institutional Review Board (#5404), and London School of Hygiene & Tropical Medicine ethics committee (#5266).

### Study context

Ethnographic fieldwork was undertaken in rural district within an hour's drive from Kisumu, the provincial capital of Nyanza where the KEMRI/CDC main offices are situated. Health indicators are poor in this area, with infant and under-five mortality rates of 25/1,000 live births and 227/1,000 live births respectively.^15^ HIV is twice as prevalent as the national average of 7.1%^16^ and, in spite of the successful implementation of an HIV care and treatment programme, AIDS related mortality and suffering affects almost every family in the area.^17^ Inhabitants of Siaya district mainly engage in farming, fishing and petty trading, and it is estimated that 64–74% live below the poverty level.^18^

## Findings

Our findings are organised under two main headings in order to explore the emerging concepts with relation to institutional considerations and VRs personal perspectives and experiences.

### Institutional framing of the role of VRs

‘…we also have a system, a VR system, they are very resourceful, we use them to capture the feelings at the community, at every village level.’ (Researcher, 01)

Since KEMRI's establishment in 1979, and inspired by contemporary ideals of Alma Ata, village volunteers have played a critical role in its activities. Initially, voluntary ‘village health helpers’ acted as agents of change to promote health development through community-initiated projects. However, as KEMRI's portfolio expanded, trials began to be conducted separately from community-led projects. In collaboration with CDC, KEMRI developed extensive research infrastructures in western Kenya. This collaboration is formally referred to as the KEMRI/CDC Research and Public Health Collaboration and it accounts for a substantial part of the KEMRI research programme. In the areas where we conducted our fieldwork community members often referred to this collaboration as CDC.

With the expansion of the research programme a clear demarcation between the practice and social organisation of science, and regular health services and community activities became apparent. With this, the role of village volunteers within KEMRI/CDC evolved from being agents of change to becoming facilitators of research. Their involvement was formalised with the establishment of a health and demographic surveillance system (HDSS) in 2001. At this point they became referred to as ‘village reporters’, and a standard operating procedure (SOP) was developed to define their role.

This SOP (Text Box 1) states that VRs represent the ‘interface’ between the community and KEMRI/CDC staff. Hence the nature of their work is bi-directional and challenges can arise in their interactions with community members and researchers. The term ‘interface’ implicitly reflects a gap between the practice of science and community experience. VRs are seen as those who can cross this boundary and create inroads which will facilitate research implementation. They can also provide insights about the nature of this boundary and its implications for practice. As the quote at the start of this section implies VRs are viewed as the backbone of the CE programme at KEMRI/CDC.

**Text Box 1. Definition of Village Reporter**

‘A Village Reporter (VR) is an individual selected by the community members after meeting specified criteria (see below), to support the implementation of KEMRI/CDC projects and studies. This individual is the interface between the community and the KEMRI/CDC staff. The VR will support all KEMRI/CDC projects in the designated geographic area. The support offered by VRs is an essential and valued component to the success of our work. The village reporters are not permanent employees. They are engaged by projects on a need basis and are paid centrally according to how many days/hours they worked.’

Hiring Criteria for VRs:

- Be respected members of the community
- Able to read and write
- Have basic knowledge of public health
- Willing and ready to work

KEMRI/CDC Standard Operating Procedure No 11, version 1^st^ November 2011.

Formalising the role of VRs in 2001 led to changes in the range and type of people engaged. Earlier trials mainly involved traditional birth attendants, typically older women, who had benefitted from additional training from governmental and non-governmental organisations. The new selection procedures resulted in a broader representation across age and to a certain extent gender. People interested in becoming VRs are nominated by their villages according to criteria provided by KEMRI/CDC (see Text Box 1). Their selection is endorsed by village elders and administrative chiefs, ‘because it is now again upon us to identify good people for the CDC to be VRs’ (Assistant Chief, 56). VRs’ main responsibilities are to record births and deaths for the HDSS and provide this data at weekly meetings. Trials also involve VRs in mobilisation, identifying and following up participants. VRs are primarily accountable to the senior KEMRI/CDC community liaison officer, but they also report to HDSS field supervisors, trial supervisors and trial-specific community liaison staff. By 2011 there were 414 VRs working in the health and demographic surveillance area; 171 (131 women, 40 men) of these are based in the area where this study was conducted.

VRs conduct their duties alongside other activities; many volunteer for other organisations and most are small-scale farmers. They belong to a growing social group who seek out as many informal employment positions as possible to make a living and support their families. As VRs, they are paid fixed fees for providing demographic data, and flat daily or hourly rates for other activities. On average they can earn 3200 Kenyan Shillings (KES) (US$35.11) per month, which is the equivalent of a labourer or watchman in the city of Kisumu, and a substantial addition to their other earnings – in particular as it does not involve relocation. In addition, next-of-kin can access a funeral allowance of KES 2000 (US$21.94) if a VR dies. (This allowance was authorized at a time when there was limited access to antiretroviral therapy, a significant consideration given the heightened prevalence of HIV, noted above.)

### VRs’ perspectives and experiences

#### The value of their approach

VRs describe themselves as ‘the link for the CDC and the village’ (VR, 8) and perceive themselves as being fundamental to CE. They argue that their involvement has helped people feel that ‘the work that is going to be done here is part of the community’ (VR Group Discussion). VRs describe themselves as living in close-knit ‘communities’ which encompass their village, the people they live with, and those they work alongside with or with whom they share a water point or school. They believe that the value of their approach lies in their close attachment to community members. They are trusted and can act as interlocutors and arbiters in ‘*opening the way*’ for research.

‘Yeah, the approach to the community, there is a little difference… because you are a villager and you are VR, so if you take the message to them, somehow they understand it better, and feel part of it, and it is different from, if the staff, project staffs, they come by themselves.’ (VR, 4)

‘You may see sometimes that a fieldworker comes to a certain home, they refuse him and then send someone to me, that those people, your people came here and I chased them away, come and explain to me what they wanted.’ (VR, 6)

VRs argue for closer collaboration between researchers, fieldworkers (i.e. employed community-based research assistants) and VRs and stress the importance of their role in encouraging community members to take part in research.

‘They take part because they know us, they know that they will get free treatment … and they see that if the person within the community, if the village reporter has just informed them about the research going on, they say, no this is a good thing [if they hear it from you?]. Yeah, this is a good thing not a bad thing (because they trust you or?). Yeah, they trust us and they know that the kind of work we are doing is a good work. So they say no, I have to enter.’ (VR, 8)

#### Balancing allegiances

VRs describe themselves as the ‘mouthpiece’ of KEMRI/CDC and the ‘mouthpiece’ of their village. Balancing these dual allegiances is complex, and in practice VRs tend to align themselves more closely with the research programme.

‘I welcome all the research now, I only tell the people in my village the good that I have seen in being in the study, so I tell the villagers that if you might think that your child might be sold, then even ours are in the study, so and so's child is also in the study, so it is something that has been brought to help us, not to harm us.’ (VR, 4)

‘… these people need village reporters who can answer them correctly … but if you are weak they can push you towards the wall and you cannot know how to answer them [they can give you a hard time?]. But we don't want to tarnish the work of CDC down we want to uplift it and make it different with other organizations.’ (VR, 8)

VRs are proactive in their support of research and use their own personal experience to offset rumours and underline the benefits of research participation. They lend less weight to the disadvantages and potential risks of taking part in trials-an important matter which raises concerns about undue influence. It is also evident that VRs clearly take great pride in their association with KEMRI/CDC and are keen to uphold the reputation of the organisation. While not ethically problematic per se this does suggest that VRs’ balance of representation tends to favour the scientific rather than the community perspective.

#### Managing community expectations

An additional complexity arises out of VRs’ close proximity, in terms of kinship as well as geography, to trial participants and villagers. While a key strength, such attachment can also be a major challenge for VRs personally. VRs are members of a ‘partrilineal-virilocal’ society wherein people live with their kin in clan-based areas. In this culture sharing and taking care of one's kin is extremely highly valued. VRs’ association with KEMRI/CDC raises implicit and explicit expectations relating to the provision of concrete practical and financial assistance to trial participants. The case study in Text Box 2 is characteristic of the pressures that VRs have to negotiate in their role as CI.

**Text Box 2. Negotiating Expectations: Recollections of a Home Visit**

The VR accompanied a group of researchers (2 expatriates & 1 Kenyan) to a household chosen at random to participate in a survey.

‘When they arrived there was only one mama who was very old, ninety something years old and she didn't have something to eat, she didn't have something to wear, she was old desperate and poor and the same time she was sick. What they did, they just took blood, and she was staying with her grandson, so after taking blood, she was given some medicine. Was it medicine? I think some tablets. Again we went to look for her grandchild who was working, trying to train as a carpenter, next to the prison, so we brought him back, asked him some questions, also we took blood from his finger tips and we went away with those wazungu (white people). So people were asking me, the state of that woman, could we just even just give her ten shillings or fifty shillings, and we have walked with two wazungu and someone who is senior in CDC, we just take blood from this woman, we know she anaemic, she don't have something to eat, she is desperate and poor, you just take blood and go away that you are doing research on malaria. Is it normal surely, if a muzungu (a white person) can come to a house like that, that house was pathetic, a grass thatched house that is leaking. So I had a lot of hard time after that because that woman was too old, too poor, too desperate and was living in a compound alone. That grandson used to come at night and go out at dawn, so they asked me why don't you even just give one hundred shillings to that woman for a kilo of sugar, of unga (flour).’ (VR, 6)

#### Motivation and involvement in trials

Becoming a VR is described as a significant event: ‘We were like five people and people queued behind us, that is the way we were elected, not nominated really, but elected by the community’ (VR, 6). Election earns VRs significant respect at community level, and improves their status financially and socially.

‘The best thing of being a VR … even though it's a voluntary service, but I see that it's not voluntary because now, at the beginning, I didn't know where the bank was, even though I knew where it was, but I happened not to have entered in. Our leader, the community liaison officer, had made efforts, we are now having the ATM cards that I could have not got if it was not for the being a VR. Another thing is that I might talk to a white person like you which I might not talk to if not being a VR, another thing is that I am now known everywhere by all tribes, and I know how to interact with the people, I know how to make my PR (public relations) to be better. …’ (VR, 5)

VRs highly value the ‘exposure’ they gain through being connected to KEMRI/CDC in terms of training, public relations, meeting people and accessing resources. Indeed this ‘exposure’ outweighs some of the disadvantages they associated with the informal nature of their work. In this regard, VRs described their activities as ‘partly volunteering and partly working’. Whilst they were grateful for the opportunity to work they believed that their efforts were not adequately reflected in their remuneration. Questions of pay are portrayed as having a knock-on effect impact on community members’ views of research and VRs’ relationships with researchers.

‘So first, I think when KEMRI/CDC wants to change the whole thing and to affect [mmm], the village positively in a big way, [mmm] or in a good way, they need to adjust that payment for the VRs so that we may be motivated [mmm], yah, because the cost of life is going high … such things also can maybe interfere with some little things in the research.’ (VR, 6)

VRs also find it difficult to reconcile inconsistencies in their terms engagement and their differing levels of involvement in individual trials.

‘Ok, the real issue is that with the KEMRI/CDC is like we have so many projects, and the way they pay their people is different. Like now you can see that Trial C is like is making use of so many funds like once you are taken to the training you are given refreshments, and again the type of, the number of days that they give you [mmm], are long. Now you will find the Trial B people, they take you to the training, there is no refreshments that you are given [mmm], and the number of days for mobilization that you are given is less [ok], so that's why they were complaining.’ (VR, 5)

It is trial investigators’ prerogative to determine how they will involve VRs based on protocol requirements and related plans for CE and recruitment. VRs core function is to assist with ‘community mobilisation'-which can be variously interpreted as gathering people together, raising awareness, to more direct involvement in the recruitment of participants. In Trial C for example, VRs were asked to identify potential participants and take FWs to their compounds. VRs accordingly played a significant direct role in recruitment. Less directly, VRs in Trial A were asked to tell mothers with newborns about the study and to advise them to visit their local health facility to learn more. By contrast, Trial B researchers were more reluctant to involve VRs in distributing trial information. They questioned VRs’ capacity to explain the content of a recruitment brochure. Hence VRs were simply asked to raise awareness about the trial.

VRs feel more positive about trials which involve them closely in trial activities. Limited involvement in trials disheartened them and made them less proactive in their support. At the start of Trial B, research staff became concerned that VRs were not promoting their trial and even communicating negative attitudes at village level. In a group discussion VRs acknowledged that they and some of their peers had felt side-lined by their limited role in Trial B. This had affected their morale and led to the indifference which had alarmed researchers. These dynamics illustrate the influence horizontal and vertical power relations can have on the conduct of research.

## Discussion

VRs stress their unique approach, positioning and invaluable role in CE and trial implementation. How they describe these is reminiscent of concepts presented in the literature on relational ethics.^19^ VRs value their work with KEMRI/CDC and hold the trust of many people in the places where they live and work. Researchers also recognise VRs central importance to their work with local communities. But, as this paper has shown, challenges can arise from trust, attachment and relationships with researchers which must be recognised, understood, and properly addressed in order to realise the full positive potential of community intermediaries such as VRs.

Managing villagers’ expectations of concrete assistance is a challenge related to the concept of attachment. VRs’ physical, familial and cultural proximity to trial participants places them in a difficult position of having to negotiate implicit and explicit expectations of help in a cultural setting in which sharing and mutuality remain cherished – if not necessarily obeyed-moral imperatives.^20^ Their relationships as clan members, friends or neighbours require them to respond personally, and to explain why KEMRI/CDC cannot provide help with more basic needs such as clothing and food. Interestingly, in giving such explanations, VRs talk about how help could be construed as ‘coercive’, paraphrasing the concerns with ‘undue inducement’ voiced by ethics guidelines, to which they were exposed during initial training and orientation. VRs and similar community intermediaries need further support and guidance from researchers on how to negotiate these kinds of expectations.

When it comes competing allegiances it is clear that VRs have become closely aligned with KEMRI/CDC. This can compromise their impartiality in the promotion of trials and blur the lines between trust, close relationships and undue influence. In reverse, our findings also show how low morale can affect the way VRs relay information about trials within their communities. VRs who feel dissatisfied about their involvement in specific trials can be passive towards, or even influence opinion against such trials. This is another form of misuse of power relations which bears similarities with the ‘horizontal and vertical exploitation’ described by Simon & Mosavel^21^ and Landy & Sharp.^22^ VRs in this case are controlling their social networks and making it more difficult for researchers to benefit from them. Partly, this phenomenon stems from their remuneration system (according to work done); partly, it depends on how far VRs feel their contribution is recognised and appreciated. To address this challenge VRs working conditions need to be reconsidered and dialogue between researchers and VRs strengthened. VRs and researchers are jointly responsible for identifying working practices which will support ethical research and minimise the potential for vertical and horizontal exploitation.

Our findings suggest that we need to develop our thinking about the more problematic aspects of relational ethics which can arise in the implementation of health research. The challenges identified warrant more in-depth examination than we were able to give them within the context of our broader study on CE. However the fact that VRs face these challenges suggests that they are engaging with their communities and the KEMRI/CDC research programme at a deep level. To strengthen this work KEMRI/CDC has started to foster closer working relationships and improved communication between trial teams and VRs. They have also adopted a more consistent approach to the involvement of VRs in different research projects. These important first steps will go some way to guaranteeing that the advantages of the ‘VR system’ outweigh potential disadvantages. To advance even further, participatory deliberation and related decision-making about VRs working practices and conditions, and their rights and responsibilities is required. We acknowledge that this is not a straightforward undertaking, but we believe that it could represent a critical investment for the continuity of positive and productive relationships between researchers and local communities.

## Footnotes

1 World Health Organisation (1978) *Declaration of Alma Ata: International Conference on Primary Health Care, Alma-Ata, USSR, 6–12 September*. Geneva: WHO. Available at: http://www.searo.who.int/LinkFiles/Health_Systems_declaration_almaata.pdf [Accessed 20^th^ June 2012].

2 C.O. Oyaya & S.B. Rifkin. Health Sector Reforms in Kenya: An Examination of District Level Planning. *Health Policy*. 2003; 64(1): 113–127; J. Rohde, M. Chatterjee & D.E. Morley (eds) 1993. *Reaching Health for All*. Oxford: Oxford University Press.

3 Republic of Kenya Ministry of Health. 2006 *Taking the Kenya Essential Package for Health to the Community: A Strategy for the Delivery of Level One Services*. Nairobi: Ministry of Health. Available at: http://marsgroupkenya.org/pdfs/2011/01/Ministry_PDFS/Ministry_of_Public_Health_and_Sanitation/Documents/Taking_the_Kenya_Essential_Package_for_Health_to_the_Community.pdf [Accessed 20 June 2012].

4 S.R. Benatar & P.A. Singer. A New Look at International Research Ethics. *BMJ*. 2000; 3221: 824–826; S.R. Benatar & P.A. Singer. Responsibilities in International Research: A New Look Revisited. *J Med Ethics* 2010; 36(4): 194–197; E.J. Emanuel et al. What Makes Clinical Research in Developing Countries Ethical? The Benchmarks of Ethical Research. *J Infect Dis* 2004; 189: 932–937.

5 E.J. Emanuel et- al., op cit note 4, pp. 932–937.

6 P.O. Tindana et- al. Grand Challenges in Global Health: Community Engagement in Research in Developing Countries. *PLoS Med* 2007; 49: 1451–1455.

7 E.J. Emanuel et al., op cit note 4, pp. 932–937.

8 P.W. Geissler et al. ‘He is now like a Brother, I can even give him some Blood’-Relational Ethics and Material Exchanges in a Malaria Vaccine ‘Trial Community’ in The Gambia. *Soc Sci &Med* 2008; 696–707.

9 L. Gilson. Trust and Health Care as a Social Institution. *Soc Sci & Med.* 2003; 56(67): 1452–1468.

10 C.S. Molyneux, N. Peshu & K. Marsh. Trust and Informed Consent: Insights from Community Members on the Kenyan coast. *Soc Sci & Med*. 2005; 61(7): 1463–1473; C.S. Molyneux et al. ‘Even if They ask You to Stand by a Tree all Day, You will Have to Do it (Laughter)…!’: Community Voices on the Notion and Practice of Informed Consent for Biomedical Research in Developing Countries. *Soc Sci & Med*. 2005; 61(2): 443–454.

11 C. Gikonyo et al. Taking Social Relationships Seriously: Lessons Learned from the Informed Consent Practices of a Vaccine Trial on the Kenyan Coast. *Soc Sci Med.* 2008; 67(5): 708–720.

12 C. Simon & M. Mosavel. Community Members as Recruiters of Human Subjects: Ethical Considerations. *Am J Bioeth.* 2010; 10(3): 3–11.

13 D.C. Landy & R.R. Sharp. Examining the Potential for Exploitation by Local Intermediaries. *Am J Bioeth*. 2010; 10(3): 12–13.

14 D. Silverman. 2005. *Doing Qualitative Research*. London: Sage Publications.

15 K. Adazu et al. Health and Demographic Surveillance in Rural Western Kenya: A Platform for Evaluating Interventions to reduce Morbidity and Mortality from Infectious Diseases. *Am J Trop Med Hyg* 2005; 73(6): 1151–1158.

16 Kenya National AIDS and STI Control Programme (NASCOP). 2009 Kenya AIDS Indicator Survey 2007: Final report. Nairobi: NASCOP.

17 R.J. Prince & P.W. Geissler. 2010. *‘The Land is Dying’ Contingency, Creativity and Conflict in Western Kenya*. Oxford, New York: Berghahn.

18 Kenya Central Bureau of Statistics. 2005. *Geographic Dimensions of Well-Being in Kenya: Who are Where are the Poor? A Constituency Level Profile*. Nairobi: Central Bureau of Statistics.

19 C. Gikonyo et al. *op cit*. note 12. pp. 708–720; C.S. Molyneux, N. Peshu & K. Marsh. *op cit.* note 11. pp. 443–454; P.W. Geissler et al. *op cit*. note 9. pp. 696–707.

20 R.J. Prince & P.W. Geissler. *op cit.* note 18.

21 C. Simon & M. Mosavel. *op cit.* note 13. pp. 3–11.

22 D.C. Landy & R.R. Sharp *op cit*. note 14. pp. 12–13.

